# Musculoskeletal Disorders: From Molecular Pathology to Novel Therapeutic Approach

**DOI:** 10.3390/ijms27188098

**Published:** 2026-09-11

**Authors:** María Orosia Lucha-López

**Affiliations:** Unidad de Investigación en Fisioterapia, Spin off Centro Clínico OMT-E Fisioterapia SLP, Universidad de Zaragoza, C/Domingo Miral s/n, 50009 Zaragoza, Spain; orolucha@unizar.es; Tel.: +34-626480131

Musculoskeletal disorders are among the leading causes of disability worldwide and represent a major driver of physical therapy needs across the life course [1]. This burden is likely to grow in the coming decades as populations age, obesity and metabolic disorders remain highly prevalent, and more people live longer with chronic non-communicable diseases [1].

In this scenario, musculoskeletal research increasingly calls for perspectives that extend beyond anatomy, biomechanics, or symptom control alone. A more integrated understanding may help to better connect molecular pathology with clinical manifestations, pain mechanisms, functional impairment, tissue degeneration, and response to treatment.

The title of this Special Issue, “Musculoskeletal Disorders: From Molecular Pathology to Novel Therapeutic Approach,” reflects this integration. Musculoskeletal conditions are no longer adequately understood as isolated disorders of single tissues. This broader view is strongly represented in the articles collected in this Special Issue, which collectively address hormonal, inflammatory, metabolic, genetic, cellular, and regenerative mechanisms across different musculoskeletal disorders (Contributions 1–14).

One theme emerging from the Special Issue is the need to place local tissue pathology within systemic biological networks. The review by Losasso et al. examines the bidirectional metabolic links between metabolic-associated fatty liver disease (MAFLD) and sarcopenia (Contribution 2). MAFLD and sarcopenia appear to be closely interconnected through several shared pathophysiological mechanisms, including insulin resistance, altered skeletal muscle secretory capacity, chronic low-grade inflammation, oxidative stress, and mitochondrial dysfunction. These processes may help explain why both conditions often coexist and influence each other. From a clinical perspective, patients who present both sarcopenia and MAFLD tend to show more severe liver fibrosis than those with MAFLD alone. Interestingly, treatment aimed at sarcopenia has also been associated with clinical improvement in MAFLD, further supporting the link between these two conditions.

This systemic perspective is complemented by the systems biology approach to sarcopenia discussed by Ceyhan et al. (Contribution 4), who emphasize that the heterogeneity of sarcopenia requires integrative analyses across multiple molecular and biological layers rather than reliance on single-pathway explanations. Indeed, sarcopenia involves interacting mechanisms related to type II fiber atrophy, intramuscular fat infiltration, mitochondrial dysfunction, oxidative stress, impaired muscle regeneration, chronic inflammation, endocrine decline, anabolic resistance, neuromuscular denervation, vascular aging, and gut microbiome dysbiosis. This complexity supports the need to study sarcopenia as a multifactorial condition in which metabolic, inflammatory, hormonal, neural, vascular, regenerative, and microbiota-related pathways converge to drive muscle degeneration and functional decline. Thus, sarcopenia has evolved from a concept centered primarily on muscle mass loss to a broader skeletal muscle disorder in which muscle strength and physical performance have gained increasing importance [2], while its underlying pathophysiology is now understood as the result of multiple interacting metabolic, inflammatory, hormonal, neuromuscular, vascular, regenerative, and microbiota-related mechanisms (Contribution 4).

Cedeno-Veloz et al. further illustrate the clinical relevance of systemic inflammation by comparing inflammatory signatures between older adults with osteoporotic hip fractures and non-fractured patients with advanced hip osteoarthritis (Contribution 10). Patients with osteoporotic hip fractures showed higher systemic inflammatory activity, particularly elevated C-reactive protein and systemic immune-inflammation index, while C-reactive protein was consistently associated with lower bone mineral density across the hip, lumbar spine, and wrist. These findings support an association between systemic inflammation, osteoporosis, and fragility fracture status, while suggesting that inflammatory biomarkers may serve as complementary tools in fracture risk assessment (Contribution 10).

A second cross-cutting issue is the insufficient translation of molecular and cellular biomarkers into clinically useful stratification tools. This gap has been addressed in this Special Issue in relation to osteoarthritis, statin-induced myopathies, and temporomandibular disorders.

The global burden of osteoarthritis has increased markedly over recent decades and is projected to continue rising with population growth and aging, underscoring its importance as a major public health problem [3]. Osteoarthritis is now understood as a whole-joint disorder involving interacting pathological changes in articular cartilage, subchondral bone, synovium, and other periarticular tissues, driven by biomechanical and inflammatory processes [4]. In addition, animal models of arthritis-induced pain have helped to identify sex-related biological and molecular mechanisms underlying pain processing and modulation [5]. These differences appear to involve several molecular mechanisms, including differential contributions of immune cell populations, toll-like receptor 4 (TLR4)-dependent and TLR4-independent signaling pathways, and inflammatory cytokines [5]. Costa et al. investigate sex-related circulating microRNAs as potential biomarkers for early knee osteoarthritis (Contribution 14). Bioinformatic analyses associated these microRNAs with osteoarthritis-relevant tissues and biological processes, including cartilage, bone, bone marrow, extracellular matrix remodeling, inflammation, lipid metabolism, and intracellular signaling pathways. Among their predicted common targets, adenosine triphosphate citrate lyase (ACLY) and phosphoinositide-3-kinase regulatory subunit 1 (PIK3R1) were identified as potential molecular nodes linking the observed microRNA profile to metabolic regulation, epigenetic modulation, inflammatory responses, and the phosphoinositide 3-kinase/protein kinase B/mechanistic target of rapamycin (PI3K/AKT/mTOR) signaling pathway (Contribution 14).

Prieto-Peña et al. address a different but equally important problem: the identification of genetic biomarkers that may help distinguish statin-associated anti-HMGCR immune-mediated necrotizing myopathy from non-immune statin-associated myotoxicity (Contribution 11). The study showed that HLA-DRB111 was strongly associated with anti-HMGCR immune-mediated necrotizing myopathy but not with non-immune statin-associated myotoxicity, suggesting a phenotype-specific genetic predisposition. Among the HLA-DRB111 alleles, HLA-DRB111:01 was the only allele significantly associated with the anti-HMGCR IMNM phenotype. In contrast, the SLCO1B1 rs4149056 variant was not significantly associated with either anti-HMGCR immune-mediated necrotizing myopathy or non-immune statin-associated myotoxicity in this cohort. These findings suggest that HLA-DRB111 testing may help distinguish immune-mediated from non-immune statin-associated myopathy, although larger multicenter studies are needed to validate its clinical utility (Contribution 11).

Zieliński and Pająk-Zielińska synthesize evidence on estrogen levels and temporomandibular disorders, highlighting the biological plausibility of hormonal modulation while also underlining substantial heterogeneity in methods and analyses (Contribution 1). This updated systematic review suggests that estrogen levels are associated with pain modulation in the temporomandibular joint and the wider orofacial region, although the evidence directly linking estrogen to the occurrence of temporomandibular disorders remains inconclusive. The included studies were highly heterogeneous in terms of diagnostic criteria, hormonal assessment methods, population characteristics, and reporting of estrogen concentrations, which limited quantitative synthesis and the possibility of drawing firm conclusions. Mechanistically, estrogen may influence temporomandibular pain through estrogen receptor-mediated, inflammatory, and nociceptive pathways. Importantly, the available evidence does not indicate that either high or low estrogen levels alone consistently determine pain severity; rather, the relationship appears to depend on hormonal fluctuations and the specific physiological context. Low-estrogen states, including certain phases of the menstrual cycle and menopause, have been associated with increased temporomandibular and orofacial pain in several studies, while rapid changes in estrogen levels have also been associated with increased pain. Experimental evidence further suggests that elevated estrogen levels may enhance pain sensitivity through estrogen receptor-mediated inflammatory and nociceptive mechanisms (Contribution 1).

A third theme concerns tissue crosstalk. The musculoskeletal system functions as an integrated biological network in which muscle, bone, joint, fascia, nerve, immune cells, vascular structures, and connective tissue matrices interact continuously. This Special Issue includes several contributions that make this network perspective explicit.

Gatti et al. propose an in vitro model of crosstalk among neurons, muscle, and bone to explore mesenchymal stromal cell-derived extracellular vesicles as a potential treatment for osteosarcopenia (Contribution 9). This study developed a triple-culture in vitro model combining osteoblasts, myotubes, and neuron-like cells to investigate bone–muscle–neuron crosstalk in osteosarcopenia. Dexamethasone-treated osteoblasts with an osteoporotic-like phenotype triggered a pathological cascade affecting the muscle and neuronal compartments, including myotube atrophy, impaired neuromuscular junction formation, altered synaptic markers, and disrupted neuronal mitochondrial organization. Extracellular vesicles derived from human amniotic fluid stem cells counteracted several of these alterations, even when applied only to the neuron–muscle compartment, suggesting that their therapeutic effects may be transmitted through intercellular communication mechanisms. Overall, the findings support extracellular vesicles as potential therapeutic tools for osteosarcopenia, although in vivo validation is required before clinical translation (Contribution 9).

The study by Gatti et al. (Contribution 9) can be interpreted within the broader framework proposed by Alcaraz et al. [6], who highlighted mesenchymal stem/stromal cell-derived extracellular vesicles as promising alternatives to mesenchymal stem/stromal cell-based therapy for musculoskeletal disorders. Alcaraz et al. [6] emphasized that many regenerative and immunomodulatory effects of mesenchymal stem/stromal cells are mediated through paracrine mechanisms, particularly extracellular vesicles carrying proteins, lipids, nucleic acids, particularly microRNAs, and other bioactive components capable of regulating inflammation, angiogenesis, cell survival, differentiation, extracellular matrix remodeling, and tissue repair. In this context, Gatti et al. (Contribution 9) provide a more specific experimental contribution by showing that extracellular vesicles derived from human amniotic fluid stem cells may interfere with the pathological crosstalk among osteoblasts with an osteoporotic-like phenotype, myotubes, and neuron-like cells in an in vitro model of osteosarcopenia. Their findings extend the general therapeutic rationale reviewed by Alcaraz et al. [6] to a complex bone, muscle, nerve axis, suggesting that extracellular vesicles may not only support tissue repair locally, but also modulate intercellular communication across musculoskeletal and neuromuscular compartments.

Liang et al. review neuromuscular junction biology, highlighting the complexity of cell–cell communication, interspecies differences, and the limitations of extrapolating findings from animal models to human neuromuscular junction biology (Contribution 6). This review highlights the neuromuscular junction as a specialized synapse that mediates communication between motor neurons and skeletal muscle fibers, with dysfunction at this interface contributing to neuromuscular disease and age-related muscle decline. Rather than being a static structure, the neuromuscular junction is presented as a dynamic and plastic interface capable of remodeling in response to exercise, disuse, aging, injury, inflammation, metabolic stress, and neurodegenerative disease. The review also emphasizes that human neuromuscular junctions differ markedly from rodent neuromuscular junctions in size, presynaptic branching complexity, and postsynaptic organization, limiting the direct extrapolation of findings across species and underscoring the need for expanded human neuromuscular junction research and more representative experimental models to improve the understanding of neuromuscular pathology and therapeutic development (Contribution 6).

Wang and Zhou synthesize evidence on macrophage infiltration, activation, and therapeutic implications in skeletal muscle injury and repair, emphasizing the temporal and phenotypic heterogeneity of macrophage responses (Contribution 7). This review highlights the central role of resident macrophages and blood monocyte-derived infiltrating macrophages in the inflammatory response triggered by skeletal muscle injury. In acute muscle damage, macrophage activation follows a temporally coordinated sequence, in which early pro-inflammatory macrophages contribute to necrotic tissue clearance and muscle satellite cell activation and proliferation, whereas subsequently emerging macrophage populations contribute to inflammation resolution, myogenic differentiation and fusion, and extracellular matrix remodeling. In dystrophic muscle, particularly in mouse models of Duchenne muscular dystrophy, injury-associated macrophage populations persist in the context of chronic muscle damage, potentially contributing to sustained inflammation, progressive fibrosis, and fibrofatty muscle replacement. The authors therefore suggest that therapeutic strategies aimed at jointly suppressing inflammatory monocyte infiltration and pathological resident macrophage expansion may provide an adjunct approach for reducing macrophage-driven pathology in Duchenne muscular dystrophy (Contribution 7).

The concept of inflamm-aging provides a broader biological framework for understanding the role of macrophages in skeletal muscle pathology. Franceschi et al. [7] proposed that aging is characterized by a progressive, chronic, and subclinical pro-inflammatory state driven by the lifelong accumulation of antigenic and other forms of stress. Within this model, macrophages occupy a central position because their chronic age-related activation, termed macroph-aging, contributes to the systemic inflammatory background that increases susceptibility to age-related diseases. This perspective connects directly with the review by Wang and Zhou, in which macrophages are presented as essential regulators of skeletal muscle repair after acute injury but as potential drivers of chronic pathology when injury-associated macrophage programs persist (Contribution 7).

Zieliński’s scoping review on fascia highlights another connective-tissue dimension that is clinically relevant but methodologically challenging to study (Contribution 5). This scoping review presents fascia as a dynamic connective tissue network involved in force transmission, proprioception, extracellular matrix remodeling, inflammation, movement organization, and pain generation. The article suggests that future fascia research will increasingly focus on the myofascial unit, movement control, and chronic pain, particularly in older adults, moving beyond a muscle-centered view of musculoskeletal function. It also highlights the growing relevance of modern imaging techniques for detecting fascial alterations and the future investigation of therapeutic approaches such as manual therapy, fascial manipulation, stretching, and functional training. Other interventions, including yoga, acupuncture, and tai chi, have also emerged as topics of interest in fascia and pain research. Together, these developments may provide new insights into the diagnosis and treatment of fascial dysfunctions (Contribution 5).

This Special Issue also illustrates the importance of experimental models while acknowledging their translational limitations.

Sánchez-Duarte et al. provide preclinical evidence that apocynin may mitigate diabetic muscle atrophy by reducing intramuscular triglyceride accumulation and oxidative stress (Contribution 8). Sánchez-Duarte et al. showed that streptozotocin-induced diabetes produces a diabetic myopathy phenotype characterized by hyperglycemia, body weight loss, dyslipidemia, reduced skeletal muscle weight, decreased muscle fiber cross-sectional area, increased lipid peroxidation, intramuscular triglyceride accumulation in fast-twitch muscle, and upregulation of atrophy-related genes. Apocynin treatment partially counteracted these alterations by reducing fasting blood glucose levels, improving body weight, and exerting hypolipidemic effects through reductions in serum triglycerides, total cholesterol, and very low-density lipoprotein cholesterol. At the skeletal muscle level, apocynin increased extensor digitorum longus and soleus muscle weight, reduced intramuscular triglyceride accumulation in the extensor digitorum longus, and decreased lipid peroxidation in both fast- and slow-twitch muscles. These effects were accompanied by muscle-specific changes in the expression of regulators of muscle atrophy and homeostasis, including downregulation of muscle RING-finger protein-1, atrogin-1, and myostatin, together with upregulation of follistatin and peroxisome proliferator-activated receptor gamma coactivator 1-alpha. Overall, the findings suggest that apocynin mitigates diabetic muscle atrophy through effects involving antioxidant, hypolipidemic, and anti-atrophic pathways, although further studies, including protein-level validation, are required to substantiate the underlying mechanisms and therapeutic potential (Contribution 8).

Tort et al. examine hydrogen-rich water as an adjuvant capable of potentiating cannabinoid- and gabapentinoid-induced antinociceptive effects in a male mouse model of neuropathic pain (Contribution 12). Hydrogen-rich water, JWH-133 (chemically known as (6aR,10aR)-3-(1,1-dimethylbutyl)-6a,7,10,10a-tetrahydro-6,6,9-trimethyl-6H-dibenzo[b,d]pyran), and pregabalin each reduced mechanical allodynia, thermal hyperalgesia, and cold allodynia in a dose-dependent manner in mice with neuropathic pain induced by chronic constriction injury of the sciatic nerve. Low-dose hydrogen-rich water potentiated the antinociceptive effects of JWH-133, a selective cannabinoid receptor type 2 agonist, producing greater inhibition of neuropathic pain-like behaviors than either treatment alone. Hydrogen-rich water also enhanced the antinociceptive effects of pregabalin, with especially marked improvement in mechanical allodynia. The treatments also modulated molecular alterations in the dorsal root ganglia associated with oxidative stress, inflammation, maladaptive synaptic plasticity, and nociceptive signaling, including normalization of injury-associated increases in 4-HNE, NLRP3, and p-ERK1/2, while p-AKT showed a more complex treatment-dependent response. The findings support hydrogen-rich water as a promising adjuvant strategy for neuropathic pain therapy, although further studies are needed to address sex differences, long-term effects and safety, oral bioavailability, dosing equivalence, and clinical translation (Contribution 12).

Nakano et al. report enhanced bone regeneration using scaffold-free three-dimensional constructs of human dental pulp stem cells in a rat mandibular defect model (Contribution 13). Nakano et al. evaluated a scaffold-free bone regenerative strategy based on human dental pulp stem cells assembled into three-dimensional constructs using Bio three-dimensional printing. In this approach, dental pulp stem cells isolated from extracted human third molars were first aggregated into spheroids and then layered onto a microneedle array, where they fused into cohesive three-dimensional cellular constructs without the use of biomaterial scaffolds. This methodology allowed the generation of transplantable structures composed exclusively of human dental pulp stem cells, thereby eliminating potential confounding effects associated with scaffold materials while preserving a defined three-dimensional architecture. Compared with monolayer cultures, the spheroids showed increased expression of stemness-related, early osteogenic, and osteogenic-predictive markers. When transplanted into critical-sized mandibular defects in immunodeficient rats, these scaffold-free constructs significantly enhanced bone healing compared with non-transplanted controls, with increased bone volume and bone mineral content on micro-computed tomography. Histological and immunohistochemical analyses further confirmed newly formed bone containing both host-derived cells and transplanted human dental pulp stem cell-derived cells, suggesting that bone regeneration may involve direct cellular participation together with possible indirect paracrine effects. Overall, the study supports Bio three-dimensional printed, scaffold-free human dental pulp stem cell constructs as a promising strategy for mandibular bone regeneration, although further validation in larger bone-defect and large-animal models, including functional and mechanical assessments, is required (Contribution 13).

Finally, the review of Bryliński et al. on trace elements in joint function synthesizes a wide range of biological roles but also shows that evidence remains uneven across elements, pathways, and clinical conditions (Contribution 3). The review summarizes the current evidence on the role of trace elements in joint physiology and joint disease, emphasizing that elements such as iron, copper, manganese, zinc, selenium, boron, and silicon play important and element-specific roles in enzymatic activity, redox regulation, extracellular matrix synthesis, chondrocyte function, and bone remodeling. However, when their concentrations are altered, or when toxic elements such as cadmium, mercury, lead, and nickel accumulate, they may contribute to oxidative stress, inflammation, apoptosis, impaired collagen synthesis, cartilage degradation, and abnormal osteoblast, osteoclast, and synoviocyte activity. The article also highlights that trace elements interact with one another through synergistic or antagonistic mechanisms, which may have biological consequences and are particularly relevant to the safety and effectiveness of supplementation strategies. Overall, the review suggests that altered trace element homeostasis may be involved in the pathogenesis and progression of rheumatoid arthritis, osteoarthritis, psoriatic arthritis, ankylosing spondylitis, and systemic lupus erythematosus, while selected trace elements and trace element-based nanoparticles may represent potential therapeutic or adjunctive approaches, although further clinical studies are required to confirm their efficacy and safety (Contribution 3).

The articles in this Special Issue provide a coherent view of musculoskeletal disorders as biologically complex, systemically modulated, and therapeutically heterogeneous conditions. They move from molecular pathology to candidate biomarkers, experimental disease models, immunometabolic mechanisms, and regenerative or pharmacological approaches. Their collective message is that future therapeutic innovation will increasingly depend on integrative approaches that account for the interactions among biological pathways, disease stage, patient characteristics, and systemic context.

An important area not represented among the contributions to this Special Issue is low back pain, including the role of intervertebral disc degeneration. Low back pain represents one of the leading causes of disability worldwide [8] and imposes a substantial socioeconomic and healthcare burden [9]. Its pathophysiology is highly multifactorial, involving interactions among structural changes, intervertebral disc degeneration, inflammatory and nociceptive mechanisms, biomechanical and neuromuscular factors, central pain-processing mechanisms, and individual and psychosocial factors [10,11]. This complexity further illustrates the need for integrative approaches capable of connecting molecular and tissue-level pathology with pain mechanisms, functional impairment, patient characteristics, and therapeutic response. Future research addressing these interactions remains essential for the development of more effective and individualized strategies for the prevention and treatment of low back pain.

Taken together, the contributions of this Special Issue reinforce a central gap in the field: musculoskeletal disorders are frequently managed at the tissue or symptom level, while their pathophysiology often reflects interacting local and systemic processes that are multifactorial and biologically interconnected.

## Data Availability

No new data were created or analyzed in this study. Data sharing is not applicable to this article.

## Abbreviations

The following abbreviations are used in this manuscript:

MAFLD	Metabolic-Associated Fatty Liver Disease
ACLY	Adenosine Triphosphate Citrate Lyase
PIK3R1	Phosphoinositide-3-Kinase Regulatory Subunit 1
PI3K/AKT/mTOR	Phosphoinositide 3-Kinase/Protein Kinase B/Mechanistic Target of Rapamycin

## References

[1] Cieza A., Causey K., Kamenov K., Hanson S.W., Chatterji S., Vos T. (2020). Global Estimates of the Need for Rehabilitation Based on the Global Burden of Disease Study 2019: A Systematic Analysis for the Global Burden of Disease Study 2019. Lancet.

[2] Cruz-Jentoft A.J., Bahat G., Bauer J., Boirie Y., Bruyère O., Cederholm T., Cooper C., Landi F., Rolland Y., Sayer A.A. (2019). Sarcopenia: Revised European Consensus on Definition and Diagnosis. Age Ageing.

[3] Steinmetz J.D., Culbreth G.T., Haile L.M., Rafferty Q., Lo J., Fukutaki K.G., Cruz J.A., Smith A.E., Vollset S.E., Brooks P.M. (2023). Global, Regional, and National Burden of Osteoarthritis, 1990–2020 and Projections to 2050: A Systematic Analysis for the Global Burden of Disease Study 2021. Lancet Rheumatol..

[4] Loeser R.F., Goldring S.R., Scanzello C.R., Goldring M.B. (2012). Osteoarthritis: A Disease of the Joint as an Organ. Arthritis Rheumatol..

[5] Kim J.-R., Kim H.A. (2020). Molecular Mechanisms of Sex-Related Differences in Arthritis and Associated Pain. Int. J. Mol. Sci..

[6] Alcaraz M.J., Compañ A., Guillén M.I. (2019). Extracellular Vesicles from Mesenchymal Stem Cells as Novel Treatments for Musculoskeletal Diseases. Cells.

[7] Franceschi C., Bonafè M., Valensin S., Olivieri F., De Luca M., Ottaviani E., De Benedictis G. (2000). Inflamm-Aging: An Evolutionary Perspective on Immunosenescence. Ann. N. Y. Acad. Sci..

[8] Ferreira M.L., de Luca K., Haile L.M., Steinmetz J.D., Culbreth G.T., Cross M., Kopec J.A., Ferreira P.H., Blyth F.M., Buchbinder R. (2023). Global, Regional, and National Burden of Low Back Pain, 1990–2020, Its Attributable Risk Factors, and Projections to 2050: A Systematic Analysis of the Global Burden of Disease Study 2021. Lancet Rheumatol..

[9] Fatoye F., Gebrye T., Ryan C.G., Useh U., Mbada C. (2023). Global and Regional Estimates of Clinical and Economic Burden of Low Back Pain in High-Income Countries: A Systematic Review and Meta-Analysis. Front. Public Health.

[10] Kaneda G., Zila L., Wechsler J.T., Shafi K., Cheema K., Bae H., Kim S.D., Tuchman A., Li D., Sheyn D. (2025). What a Pain in the Back: Etiology, Diagnosis and Future Treatment Directions for Discogenic Low Back Pain. Bone Res..

[11] Ambrosio L., Schol J., Ruiz-Fernandez C., Denaro V., Vadalà G., Sakai D. (2026). Lost in Translation: Why Biologic Therapies for Intervertebral Disc Degeneration and Low Back Pain Have Not Reached the Clinic (Yet). JOR Spine.

